# Immunohistochemical Profile of p62/SQSTM1/Sequestosome-1 in Human Low- and High-Grade Intracranial Meningiomas

**DOI:** 10.1155/2024/5573892

**Published:** 2024-08-02

**Authors:** Antonio Ieni, Cristina Pizzimenti, Vincenzo Fiorentino, Mariausilia Franchina, Antonino Germanò, Giovanni Raffa, Maurizio Martini, Guido Fadda, Giovanni Tuccari

**Affiliations:** ^1^ Department of Human Pathology in Adult and Developmental Age “Gaetano Barresi” Section of Pathology University of Messina, Messina 98125, Italy; ^2^ Department of Biomedical, Dental, Morphological and Functional Imaging Sciences, Neurosurgery University of Messina, Viale Gazzi, Messina 98125, Italy

## Abstract

Among autophagic-related proteins, p62/SQSTM1/Sequestosome-1 represents a relevant actor in cellular proliferation and neoplastic growth. Although, recently, p62 expression has been analyzed in different neurodegenerative and glial neoplastic diseases, no available information have been reported in meningiomas, which have an high epidemiological relevance being the second most common category of intracranial tumors after gliomas. Generally meningiomas have a benign behavior, but their recurrence is not uncommon mainly when atypical or anaplastic varieties occur. However, intranuclear vacuoles have been ultrastructurally observed in meningiomas, and they were labelled by p62 antibodies. Therefore, in the present study, we have investigated p62 immunohistochemical pattern in a cohort of 133 cases representative of low- and high-grade meningiomas, to verify if p62 expression may be related to clinicopathological data, thus achieving a potential prognostic role. The p62 immunoexpression was frequently found in the nucleus and cytoplasm of neoplastic elements, and utilizing an intensity-distribution score, 55 (41.3%) cases were considered as high expressors while 78 (58.7%) cases were instead recorded as low expressors. Fifteen cases exhibited recurrences of the disease, 14 of which were codified as high expressors. Moreover, a direct relationship between p62 and Mib-1 immunoexpression as well as between p62 and neoplastic grade have been documented. Finally, we suggest that impaired autophagic flux with an increase in p62 expression may be involved in the activation of NRF2 also contributing in the development of recurrence in meningioma patients.

## 1. Introduction

It is well known that autophagy deregulation may occur in many pathophysiological processes, such as infection, cardiomyopathy, autoimmune and neurodegenerative disorders, and cancer [1, 2, 3, 4]. In this latter scope, it has been hypothesized that autophagy appears to be involved either in cell growth control or in tumor suppression [5, 6, 7, 8, 9, 10]. First, autophagy was considered to have a tumor-suppressive role by preventing the accumulation of toxic radicles, also modifying the genomic structures, and reducing the risk of metastasis [7]. By contrast, it has been suggested that autophagy may furnish metabolic supply to cancer cells, thus enhancing their survival and promoting metastatic process [8, 9].

**Figure 1:**
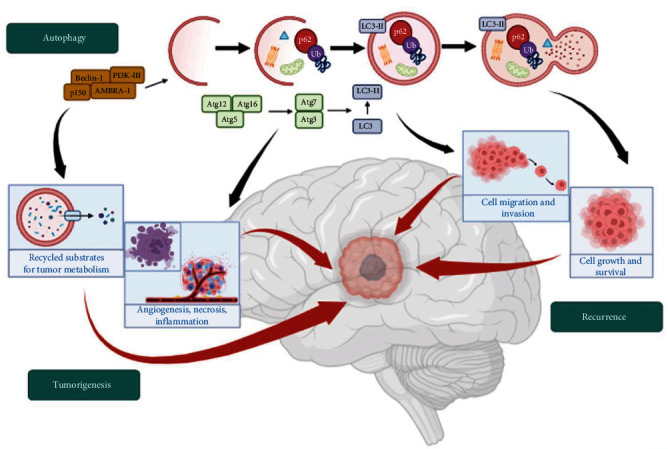
In tumorigenesis, ATGs favor the recycle of substrates for tumor metabolism sustainment and promote angiogenesis, necrosis, and inflammation, while in recurrence, they promote tumor cell survival, cell migration, and invasion.

Although various autophagy-related proteins (ATGs) influence the formation and degradation of autophagosome [11, 12, 13, 14], many other ATGs could influence tumorigenesis as well as neoplastic recurrence (Figure 1).

Specifically, p62, also known as sequestosome-1 (SQSTM1), is upregulated in human cancer [15, 16, 17, 18], exhibiting an evident expression in pancreato-biliary, mammary, and oral cancer [16, 17, 18, 19, 20]. In the central nervous system (CNS), p62 has been formerly studied in neurodegenerative disorders, such as Parkinson's and Alzheimer's diseases [21, 22, 23], while the role of p62 in gliomas is still debated [24, 25]. Nevertheless, in recent years, an increased p62 expression has been documented mainly in high grade gliomas without any relationship with isocitrate dehydrogenase (IDH) mutation status [15, 26, 27]. On the other hand, we have documented p62 immunoexpression in the nucleus and the cytoplasm of neoplastic elements in 45% of primary and 55% of recurrent cases of glioblastomas [28]. Moreover, the most favorable prognosis was appreciable when there was concordance between p62 expression and methylated status of O^6^-methylguanine-DNA methyltransferase [28].

Meningioma represents the second most common category of intracranial tumors after gliomas, generally occurring in middle-aged and elderly adults. The great majority of meningiomas are constituted by grade I neoplasm, although grade II (atypical) and grade III (anaplastic) have been recorded and associated with a worse prognosis. In fact, although meningiomas have generally a benign behavior, their recurrence is not uncommon and when it occurs, the histology may be unchanged, or it may show progression towards atypical or anaplastic varieties.

Interestingly, one of the most intriguing ultrastructural findings in meningiomas is the presence of intranuclear vacuoles, diversely labelled by p62 antibodies [29, 30]. Consequently, the presence of these intranuclear vacuoles strongly support the idea that macroautophagy takes place in meningiomas, although only ultrastructural observations have been performed in 15 cases [30], while p62 protein has been documented as possible predictor of malignancy in a series of 45 benign meningiomas, exclusively from female patients [29]. Specifically, in this latter study, signs of functioning proteosomal system can be detected using the p62 labeling in the benign, but not recurrent meningiomas [29]. Therefore, in the present paper, we have thought of interest to evaluate the immunohistochemical pattern of p62 in a large cohort of 133 low- and high-grade meningiomas, in order to verify if its expression may represent a linkage to autophagy, further revealing relationships with other clinicopathological data and achieving a prognostic role to predict the event of recurrence and/or invasion in meningiomas.

## 2. Materials and Methods

This retrospective analysis has been performed taking into consideration the Good Clinical Practice guidelines as well as the Declaration of Helsinki (revised in 2013). No informed consent was required since the nature of the study is retrospective; nevertheless, at the admission in the neurosurgical unit, all patients signed a written informed consent to utilize their biological material for scientific purposes. Patients' medical records and pathology reports are available for all cases. All samples were anonymized before histology and immunohistochemistry.

### 2.1. Case Selection

From the archives of the Department of Human Pathology of Adult and Evolutive Age (University of Messina, Messina, Italy), 133 cases of meningiomas, collected during the period 2010–2019, were selected. All patients had undergone surgical treatment in the neurosurgical unit, comprising 46 men and 87 women (mean age, 59.7 years; S.D., ±12.8 years). All cases have been histologically reviewed by two independent observers according to World Health Organization (WHO) 2021 criteria. Clinicopathological data relative to each patient (age, sex, tumor site, histology, Ki-67 labeling index, and presence of progesterone receptors) and information about time to event of recurrence were obtained from the medical records (Table 1).

The reported clinicopathological parameters as well as immunohistochemical data in relation to p62 expression are summarized in Table 1.

### 2.2. Immunohistochemistry

As extensively reported in a p62 investigation regarding gliomas [28], 4-*μ*m thick sections obtained from corresponding tissue-blocks were subjected to the immunohistochemical procedures after an adequate deparaffinization and washing in descending alcohol scale. First, slides were treated with 3% hydrogen peroxide for 10 min, and further, washed three times in deionized water and then incubated with normal sheep serum to prevent unspecific adherence of serum proteins for 30 min at room temperature. Later, sections were incubated for 30 min at 37°C with primary antihuman antisera mouse monoclonal anti-SQSTM1/p62 antibody (working dilution 1 : 200, clone 2C11, Abcam, Cambridge, UK). After three rinses with phosphate-buffered saline (PBS), sections were incubated with a biotinylated goat antirabbit IgG secondary antibody (1 : 300; Abcam, Cambridge, UK) for 20 min at room temperature and last, with horseradish peroxidase-labeled secondary antibody for 30 min and developed with diaminobenzidine tetrahydrochloride and counterstained with hematoxylin using the ULTRA Staining system (Ventana Medical Systems, Tucson, AZ, USA). The specific primary antiserum was omitted and substituted by PBS, thus achieving a negative control. The evaluation of p62 immunoreactivity was performed according to the intensity and percentage of positively stained cells, as elsewhere reported [2, 3, 4]. The cytoplasmic and nuclear immunostaining intensity was rated as follows: 0, negative; 1, weak; and 2, strong. The percentage of positively stained cells was graded as follows: grade 0, 0%–5%; grade 1, >5%–25%; grade 2, >25%–50%; grade 3, >50%–75%; and grade 4, >75%–100% for all antibodies. The intensity distribution score (ID score) was determined by adding the staining intensity score and the percentage score of positively stained cells (0–6). Meningiomas with an immunoreactive score of 0–3 were classified as negative, while positive were recorded for those with a score of 4–6. The immunohistochemical staining samples were independently evaluated by two pathologists (Antonio Ieni and Giovanni Tuccari), who were blinded to patient outcomes and other clinical findings, using a Zeiss Axioskop microscope (Carl Zeiss Microscopy GmbH, Jena, Germany) at 40x objective magnification. The interobserver agreement for p62 immunohistochemistry staining had a *κ* value ranging from 0.76 to 0.81 (substantial agreement).

### 2.3. Statistical Analysis

Statistical evaluation was performed using the SPSS version 13.0 software package (SPSS, Inc., Chicago, IL, USA). The association between p62 expression in meningiomas and clinicopathological features (age, gender, tumor site, grade, progesterone status, Ki-67 revealed by Mib-1 antibody, and recurrence) was analyzed using the Fisher exact test. Mann–Whitney test has been applied to document the possible difference between grade I and grade II–III as well as p62 immunohistochemical low and high status. A Kaplan–Meier curve comparing recurrence patterns in low and high ID scores in relation to the grade of meningioma was performed. A value less than 0.05 was considered statistically significant.

## 3. Results

In our cohort, the tumor site exhibited a various localization, and specifically the more represented ones were: convexity, 78 (58.65%); parasagittal and sagittal, 26 (19.55%); skull base, 15 (11.28%); posterior fossa, five (3.76%); intraventricular, three (2.56%); olfactory, two (1.5%); and finally, four with other different localization. Taking into consideration the grade of meningiomas, we have grouped the grade I (low; 90 cases, 69.6%) in comparison to the grade II–III (high; 43, 30.4%). In relation to progesterone receptor expression, 89 cases (66.9%) documented nuclear immunoreactivity, while 44 cases (33.1%) were constantly unreactive. Fifteen cases presented a recurrence in a temporal interval of 6–48 months (mean 20.93 and median 18), and specifically two cases were codified as grade I, whereas 13 cases were attributable to grade II–III (Table 2).

**Figure 2:**
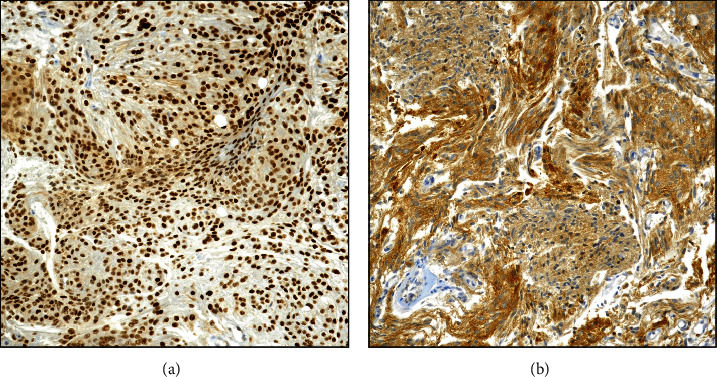
An intense, diffuse, and homogeneous immunoexpression in high-grade meningioma ((a), 300x); moderate and nonuniform staining was noted in low-grade meningioma ((b), 200x; p62 antibody, Mayer's Haemalum counterstain).

**Figure 3:**
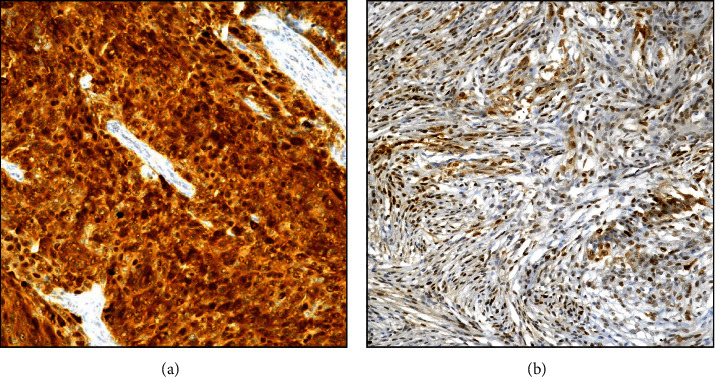
A specific nuclear immunoexpression in high-grade meningioma ((a), 200x); while a cytoplasmic localization was encountered in low-grade meningioma ((b), 200x; p62 antibody, Mayer's Haemalum counterstain).

The p62 immunoexpression was variously found in all examined meningiomas, both in the nucleus and cytoplasm of neoplastic elements (Figure 2); a peculiar exclusive nuclear or cytoplasmic immunostaining was encountered only in few cases (Figure 3). Regarding ID score, 55 (41.3%) cases were considered as high expressors (ID > 4; Figures 2(a) and 3(a)), while 78 (58.7%) cases were instead recorded as low expressors (Figures 2(b) and 3(b)). Among 15 cases exhibiting recurrences of the disease, 14/15 were codified by a high ID score, while only one case showed a lower p62 immunostaining.

In univariate analysis of meningiomas, tumor site (convexity against others; *p*=0.020), grading (*p*=0.0002), and recurrence (*p* < 0.0001) showed a significant *p* value in relation to p62 status (Table 3).

**Figure 4:**
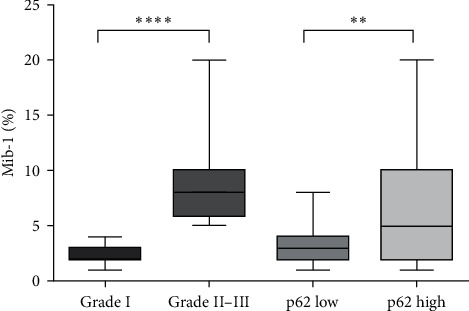
The Mann–Whitney test regarding relationships among Mib-1 percentage, tumor grade, and p62 status.  ^*∗∗∗∗*^ and  ^*∗∗*^ indicated in this figure are related to the statistical significance obtained by Mann–Whitney test in relation to grade and p62 expression encountered in different group of meningiomas.

The Mann–Whitney test documented a significant difference (*p* < 0.0001) between grade I versus grade II–III as well as between p62 high versus p62 low expression (*p*=0.0017) in relation to Mib-1 revealed percentage (Figure 4).

**Figure 5:**
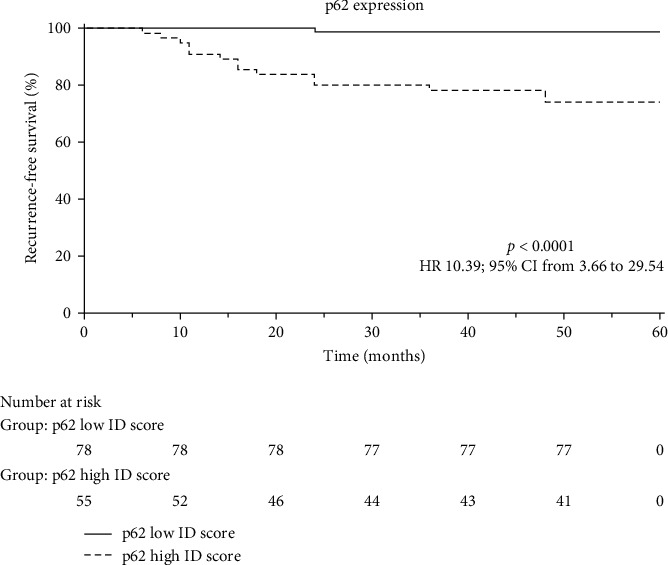
Kaplan–Meier curve showing the different behavior of p62 low and high ID score in relation to the recurrence event.

The Kaplan–Meier curve showed a significant difference (*p* < 0.0001) comparing recurrence patterns in p62 low and high ID score of meningiomas (Figure 5).

## 4. Discussion

In this study, we have analyzed the immunohistochemical expression of p62 in a cohort of meningiomas (grade I and grade II/III), determining a score for each patient and distinguishing p62 low (ID 0–3) and high expressors (ID 4–6), as elsewhere previously reported for the same autophagy-related proteins in gliomas of CNS [28]. We found a p62 immunoreaction in the nucleus and cytoplasm of neoplastic elements present in all analyzed cases of meningiomas, although a high p62 expression was found in 41.3% of cases, while a low immunohistochemical p62 profile was recorded in 58.7%. Taking into consideration the grade of meningiomas, a direct correlation between p62 immunoexpression and neoplastic differentiation was documented, since the great majority (70.78%) of grade I meningioma showed a lower p62 expression compared to grade II/III meningiomas (34.88%), with a high significant difference (*p*=0.0002). Regarding recurrence, a direct significant relationship (*p*  < 0.0001) has been detected between p62 level and the occurrence of this phenomenon; in fact, 15 cases exhibited a neoplastic recurrence, 14 of which showed a higher p62 overexpression. Applying the Mann–Whitney test stratifying data in reference to Mib-1 percentage of cases, a clear very significant distinction was made between grade I and II/III (*p* < 0.0001), with a less relevant but significant (*p*=0.0017) between p62 low and high expressors. Moreover, by Kaplan–Meier curve, a similar significant value was achieved comparing recurrence patterns in low and high p62 ID score of meningiomas.

The present study stressed a hypothesized progressive p62 enhancement moving from WHO grade I to grade II/III, as previously elsewhere suggested in gliomas [15, 26]. In detail, Deng et al. [15] have shown that overexpression of p62 endorsed glioma progression by promoting proliferation, migration, and glycolysis as well as temozolomide (TMZ) resistance and nuclear factor*-κ*B (NF-*κ*B) signalling pathway. Furthermore, p62 has been highly expressed in high-grade glioma tissues, compared with low-grade glioma tissues, mainly associated with advanced tumor stages, worse relapse-free survival (RFS) and overall survival (OS) in glioma patients [26]. Consequently, it has been suggested that p62 higher expression may stimulate classical autophagic pathway, antagonizing apoptosis and allowing the occurrence of neoplastic recurrence [17, 31, 32]. Therefore, in our opinion, p62 enhances proliferation, increases neoplastic grade, and lessens disease free survival in meningiomas.

The 2016 WHO classification of brain tumors has definitely codified three degrees of meningiomas, from the classical one to atypical and anaplastic ones [33]. Although meningiomas are generally considered a low aggressive intracranial neoplasm, it must be recorded that grade II/III should be considered really aggressive, in terms of growth fraction and local invasion, either to the dura or to the brain tissue. Therefore, in this latter group the neoplastic recurrence may represent an unpleasant event, able to influence clinical course and prognosis. In the present paper, together a direct relationship of p62 and Mib-1 immunoexpression as well as between p62 and neoplastic grade, we have documented that p62 expression is closely related to the occurrence of recurrence. Consequently, it is not surprising that in the univariate analysis, p62 status appears as strong parameter likewise to tumor grade and Mib1 expression. In the light of these data, we suggest that high p62 expression should represent an additional marker to foresee the biological behavior in meningiomas, confirming some recent findings that have demonstrated a worse prognostic behavior in glioblastoma patients, exhibiting high levels of this autophagy-related protein [28, 34, 35].

It has been underlined that autophagy also represents a mechanism of cell death that can be established even without detectable apoptosis (via autophagic death) or at the same time as apoptosis [36]. This evidence rises an intriguing question about the significance of autophagy in tumorigenesis and tumor suppression, not only in meningiomas, but also in gliomas mainly in high grade ones, in which p62 is related to worse RFS, OS, and recurrence [15, 26, 27]. Moreover, the role of autophagic proteins has also been hypothesized in other neoplasms, such as uveal melanoma and malignant pleural mesothelioma [3, 36]. Specifically, the immunoexpression of three autophagy-related proteins (Beclin-1, p62, and ATG7) has been evaluated in a series of uveal melanomas, with an overexpression of Beclin-1 that documented a relationship with histotype and better outcome; by contrast, no significant differential expression of ATG7 and p62 between patients, with or without metastasis, has been reported [3]. However, high expression of ATG7 has been considered a promising prognostic tool for patients affected by malignant mesothelioma [36]. Regarding meningiomas, the relationship between two autophagy markers, such as Beclin 1 and LC3B, with clinicopathological parameters has been previously carried out [37]. In this paper, the authors have found that expression of Beclin-1 rather than LC3B correlated to better prognosis, low pathological grade and longer survival, with a staining intensity significantly related to the pathological grade [37]. Taking into consideration the dual role of autophagy related proteins in the development of cancer, we can suggest that impaired autophagic flux with an increase in p62 protein level may be involved in the activation of NRF2 and contributes to the tumorigenesis and/or neoplastic recurrence by a more functioning proteosomal system, probably due to intrinsic neoplastic hypoxic conditions.

## Figures and Tables

**Table 1:** Clinicopathological parameters of analyzed meningiomas.

Characteristics	*n* = 133
Age, mean (±SD)	59.7 (12.8)
Gender, *n* (%)	
Male	46 (34.6)
Female	87 (65.4)
Tumor site	
Convexity	78 (58.65)
Parasagittal and sagittal	26 (19.55)
Skull base	15 (11.28)
Intraventricular	3 (2.26)
Olfactory	2 (1.5)
Posterior fossa	5 (3.76)
Others	4 (3.00)
Grade, *n* (%)	
I	90 (69.6)
II–III	43 (30.4)
Progesteron, *n* (%)	
Positive	89 (66.1)
Negative	44 (33.1)
Recurrence, *n* (%)	
Yes	15 (11.3)
No	118 (88.7)
p62, *n* (%)	
High	55 (41.3)
Low	78 (58.7)
Ki-67, *n* (%)	
≤4	90 (67.67)
>4–≤ 10	35 (26.32)
>10	8 (6.01)

**Table 2:** Age, sex, site, grade, and p62 score in 15 meningiomas showing recurrence.

Case no.	Age	Sex	Tumor site	Grading	p62 score	Recurrence (months)
1	44	M	Convexity	II	4	24
2	43	M	Posterior fossa	III	6	6
3	60	F	Convexity	III	4	16
4	40	F	Parasagittal	III	4	14
5	56	F	Convexity	II	4	48
6	60	F	Parasagittal	III	5	18
7	74	M	Convexity	II	4	48
8	68	F	Skull base	II	5	24
9	63	F	Convexity	III	5	11
10	59	F	Skull base	III	6	16
11	75	F	Convexity	II	6	11
12	76	M	Olfactory	II	4	10
13	77	F	Parasagittal	III	5	8
14	80	F	Skull base	I	6	36
15	44	F	Convexity	I	3	24

**Table 3:** Univariate analysis of prognostic parameters in examined meningiomas cases.

	Higher p62 level	Lower p62 level	*p*	OR (95% CI)
Age				
<50	12	24	0.157	1.911
≥50	43	54		From 0.8253 to 4.425
Gender				
Male	21	25	0.579	0.764
Female	34	53		From 0.3707 to 1.573
Progesteron				
Positive	33	56	0.191	0.589
Negative	22	22		From 0.2837 to 1.224
Grading				
I	27	63	**0.0002**	0.143
II–III	28	15		From 0.033 to 0.622
Recurrence				
No	1	77	**<0.0001**	26.29
Yes	14	41		3.337–207.2
Tumor site				
Convexity	26	52	**0.020**	2.400
Others	30	25		From 1.180 to 4.880

Values in bold and underline document a statistical significance.

## Data Availability

The data presented in this study are available on request from the corresponding author. The data are not publicly available due to privacy restrictions.

## References

[1] Li X., He S., Ma B. (2020). Autophagy and autophagy-related proteins in cancer. *Molecular Cancer*.

[2] Ieni A., Cardia R., Giuffrè G., Rigoli L., Caruso R. A., Tuccari G. (2019). Immunohistochemical expression of autophagy-related proteins in advanced tubular gastric adenocarcinomas and its implications. *Cancers*.

[3] Broggi G., Ieni A., Russo D. (2020). The macro-autophagy-related protein beclin-1 immunohistochemical expression correlates with tumor cell type and clinical behavior of uveal melanoma. *Frontiers in Oncology*.

[4] Ieni A., Pizzimenti C., Giuffrè G., Caruso R. A., Tuccari G. (2022). Autophagy-related prognostic signature in HER2 positive gastric carcinomas. *Current Molecular Medicine*.

[5] Eskelinen E.-L. (2011). The dual role of autophagy in cancer. *Current Opinion in Pharmacology*.

[6] Chmurska A., Matczak K., Marczak A. (2021). Two faces of autophagy in the struggle against cancer. *International Journal of Molecular Sciences*.

[7] Verma A. K., Bharti P. S., Rafat S. (2021). Autophagy paradox of cancer: role, regulation, and duality. *Oxidative Medicine and Cellular Longevity*.

[8] Gerada C., Ryan K. M. (2020). Autophagy, the innate immune response and cancer. *Molecular Oncology*.

[9] Yun C. W., Jeon J., Go G., Lee J. H., Lee S. H. (2021). The dual role of autophagy in cancer development and a therapeutic strategy for cancer by targeting autophagy. *International Journal of Molecular Sciences*.

[10] Amaravadi R. K., Kimmelman A. C., Debnath J. (2019). Targeting autophagy in cancer: recent advances and future directions. *Cancer Discovery*.

[11] Wang C.-W., Klionsky D. J. (2003). The molecular mechanism of autophagy. *Molecular Medicine*.

[12] Mizushima N., Komatsu M. (2011). Autophagy: renovation of cells and tissues. *Cell*.

[13] Feng Y., Klionsky D. J. (2017). Autophagy regulates DNA repair through SQSTM1/p62. *Autophagy*.

[14] Metur S. P., Klionsky D. J. (2021). Autophagy under construction: insights from in vitro reconstitution of autophagosome nucleation. *Autophagy*.

[15] Deng D., Luo K., Liu H. (2019). p62 acts as an oncogene and is targeted by miR-124-3p in glioma. *Cancer Cell International*.

[16] Chao X., Ni H.-M., Ding W.-X. (2022). An unexpected tumor suppressor role of SQSTM1/p62 in liver tumorigenesis. *Autophagy*.

[17] Tang J., Li Y., Xia S. (2021). Sequestosome 1/p62:a multitasker in the regulation of malignant tumor aggression (review). *International Journal of Oncology*.

[18] Li D., He C., Ye F. (2021). p62 overexpression promotes bone metastasis of lung adenocarcinoma out of LC3-dependent autophagy. *Frontiers Oncology*.

[19] Thongchot S., Vidoni C., Ferraresi A. (2021). Cancer-associated fibroblast-derived IL-6 determines unfavorable prognosis in cholangiocarcinoma by affecting autophagy-associated chemoresponse. *Cancers*.

[20] Umemura A., He F., Taniguchi K. (2016). p62, upregulated during preneoplasia, induces hepatocellular carcinogenesis by maintaining survival of stressed HCC-initiating cells. *Cancer Cell*.

[21] Haack T. B., Ignatius E., Calvo-Garrido J. (2016). Absence of the autophagy adaptor SQSTM1/p62 causes childhood-onset neurodegeneration with ataxia, dystonia, and gaze palsy. *The American Journal of Human Genetics*.

[22] Muto V., Flex E., Kupchinsky Z. (2018). Biallelic SQSTM1 mutations in early-onset, variably progressive neurodegeneration. *Neurology*.

[23] Pytte J., Anderton R. S., Flynn L. L. (2020). Association of a structural variant within the SQSTM1 gene with amyotrophic lateral sclerosis. *Neurology Genetics*.

[24] Galavotti S., Bartesaghi S., Faccenda D. (2013). The autophagy-associated factors DRAM1 and p62 regulate cell migration and invasion in glioblastoma stem cells. *Oncogene*.

[25] Chang Y.-L., Li Y. F., Chou C. H. (2021). Diosmin inhibits glioblastoma growth through inhibition of autophagic flux. *International Journal of Molecular Sciences*.

[26] Jiang T., Wu Z. (2018). Immunohistochemical assessment of autophagic protein LC3B and p62 levels in glioma patients. *International Journal Clinical Experimental Pathology*.

[27] Tamrakar S., Yashiro M., Kawashima T. (2019). Clinicopathological significance of autophagy-related proteins and its association with genetic alterations in gliomas. *Anticancer Research*.

[28] Ieni A., Pizzimenti C., Broggi G. (2022). Immunoexpression of p62/SQSTM1/sequestosome-1 in human primary and recurrent IDH1/2 wild-type glioblastoma: a pilot study. *Oncology Letters*.

[29] Kärjä V., Alafuzoff I. (2006). Protein p62 common in invaginations in benign meningiomas—a possible predictor of malignancy. *Clinical Neuropathology*.

[30] Jaskólski D., Papierz T., Liberski P. P., Sikorska B. (2012). Ultrastructure of meningiomas: autophagy is involved in the pathogenesis of intranuclear vacuoles. *Folia Neuropathologica*.

[31] Zeng R.-X., Zhang Y.-B., Fan Y., Wu G.-L. (2014). Wu, p62/SQSTM1 is involved in caspase-8 associated cell death induced by proteasome inhibitor MG132 in U87MG cells. *Cell Biology International*.

[32] Ivankovic D., Chau K.-Y., Schapira A. H. V., Gegg M. E. (2016). Mitochondrial and lysosomal biogenesis are activated following PINK1/parkin-mediated mitophagy. *Journal of Neurochemistry*.

[33] Louis D. N., Perry A., Reifenberger G. (2016). The 2016 world health organization classification of tumors of the central nervous system: a summary. *Acta Neuropathologica*.

[34] Wang Q. W., Liu H. J., Zhao Z. (2020). Prognostic correlation of autophagy-related gene expression-based risk signature in patients with glioblastoma. *OncoTargets and Therapy*.

[35] Pizzimenti C., Fiorentino V., Franchina M. (2023). Autophagic-related proteins in brain gliomas: role, mechanisms, and targeting agents. *Cancers*.

[36] Rapisarda V., Broggi G., Caltabiano R. (2021). ATG7 immunohistochemical expression in malignant pleural mesothelioma. A preliminary report. *Histology and Histopathology*.

[37] Kuo K.-L., Chen C.-H., Chen H.-I., Chung Y.-Y., Chai C.-Y. (2019). Higher expression of beclin 1 in human meningiomas is related to better clinical outcome and pathological grade. *Acta Pathologica Microbiologica Scandinavica*.

